# Sustainable Pretreatment of Food Waste for Enhanced Bioethanol Production and Improved Waste Management: A Review

**DOI:** 10.1002/fsn3.71506

**Published:** 2026-01-30

**Authors:** Shaina Sharma, Sudarshan Sahu, Gursharan Singh, Shailendra Kumar Arya, Arulazhagan Pugazhendi, Ratih Setyaningrum, Karthikeyan Ravi, Sasikala Chinnappan, Ravishankar Ram Mani, Soon Woong Chang, Balasubramani Ravindran

**Affiliations:** ^1^ Department of Biotechnology Engineering University Institute of Engineering & Technology, Panjab University Chandigarh India; ^2^ Department of Medical Laboratory Sciences Lovely Professional University Phagwara Punjab India; ^3^ Department of Marine Biology, Faculty of Marine Sciences King Abdulaziz University Jeddah Saudi Arabia; ^4^ Center of Excellence in Environmental Studies King Abdulaziz University Jeddah Saudi Arabia; ^5^ Industrial Engineering Department of Engineering Faculty Universitas Dian Nuswantoro Semarang City Indonesia; ^6^ Centre for Herbal Pharmacology and Environmental Sustainability Chettinad Hospital and Research Institute, Chettinad Academy of Research and Education Kelambakkam Tamil Nadu India; ^7^ Department of Pharmaceutical Biology, Faculty of Pharmaceutical Sciences UCSI University Cheras, Kuala Lumpur Malaysia; ^8^ Department of Civil & Energy System Engineering Kyonggi University Suwon‐si Gyeonggi‐do South Korea; ^9^ Department of Microbiology, Faculty of Arts Science Commerce and Management Karpagam Academy of Higher Education Coimbatore Tamil Nadu India

**Keywords:** bioethanol, food waste, pretreatment, sustainability, waste management

## Abstract

Rapidly increasing global food‐waste generation poses major environmental, economic, and waste‐management challenges due to its high organic load and improper disposal practices. Addressing this problem requires sustainable valorization strategies, including bioethanol production, which can simultaneously reduce waste burdens and contribute to renewable‐energy generation. This review synthesizes current knowledge on the physical and chemical characteristics of food waste, the rationale behind pretreatment methods, and their role in improving downstream bioconversion efficiency. Pretreatments—physical, chemical, physicochemical, and biological—are examined with emphasis on how they enhance hydrolysis and improve fermentable‐sugar release. Fermentation is the critical biochemical step in this pathway, as it converts the hydrolyzed sugars into bioethanol through the metabolic activity of yeast and bacteria. Enzymatic hydrolysis and microbial fermentation, the core steps that convert complex biomass into ethanol, are critically evaluated alongside bioprocessing strategies such as SHF, SSF, SSCF, and consolidated bioprocessing. The review identifies that physical and chemical pretreatments improve fermentable‐sugar release but may involve higher energy or chemical inputs, whereas enzymatic and biological methods offer more sustainable alternatives with lower inhibitory by‐product formation. Among bioprocessing strategies, SSF and SSCF consistently demonstrate higher bioethanol yields and reduced processing time compared with SHF. Consolidated bioprocessing shows strong potential for future development due to its reduced operational steps and lower overall costs. Collectively, these findings highlight the importance of integrating efficient pretreatment with optimized fermentation strategies to maximize bioethanol production while enhancing the sustainability of food‐waste management.

## Graphical abstract

The graphical abstract illustrates the conversion of food waste biomass into bioethanol through integrated pretreatment strategies. Physical (mechanical, thermal, sonication), chemical (alkali, acidic, oxidative/ROS), and biological (fungal, bacterial, enzymatic) pretreatments enhance biomass breakdown. The pretreated biomass is subsequently fermented to produce bioethanol.

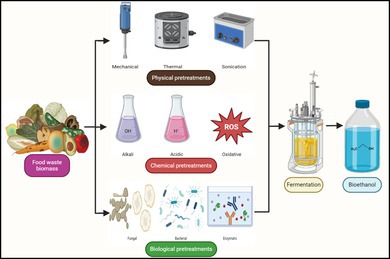

## 1 Introduction

Rapidly increasing global food‐waste generation has become a major environmental, economic, and waste‐management challenge. The Food and Agriculture Organization (FAO) estimates that 1.3–1.6 billion tons of food are wasted annually, amounting to nearly one‐third of all food produced for human consumption (FAO 2018). Per‐capita waste generation varies significantly across regions: households in Europe and North America discard approximately 95–115 kg per person per year (Daskalopoulos et al. 1998; Chandrappa and Das 2024), whereas households in Sub‐Saharan Africa and South/Southeast Asia typically generate only 6–11 kg per person annually due to differences in food systems, infrastructure, and consumption patterns (Oelofse and Nahman 2013; Oelofse et al. 2018; Rajaram and van Ginkel 2019). Asia remains the largest contributor in absolute volume, with countries such as China and India producing several hundred million tons of food waste each year (Ghosh et al. 2016; Ong et al. 2018). These rising levels of food waste impose substantial environmental and economic burdens, including greenhouse‐gas emissions, resource depletion, and escalating waste‐management costs.

A distinction must be made between food losses and food waste to contextualize this challenge accurately. Food losses occur during harvesting, post‐harvest handling, storage, and processing due to spoilage, poor infrastructure, or inefficiencies within the supply chain (Kaur and Watson 2024). In contrast, food waste refers to edible material discarded at the retail, food‐service, and consumer levels. Post‐consumer food waste is typically wet and characterized by very high moisture content, which accelerates microbial degradation, generates leachate, releases unpleasant odors, and increases methane emissions when disposed of in landfills (Amradi et al. 2023). This moisture‐rich fraction consequently poses significant environmental risks and highlights the need for sustainable alternatives to conventional disposal practices.

Food waste is categorized as a second‐generation (2G) bioethanol feedstock, as it is derived from non‐edible residues and does not compete with food crops, unlike first‐generation feedstocks such as corn or sugarcane (Igwebuike et al. 2024). Compared with lignocellulosic agricultural residues, food waste typically contains higher proportions of readily biodegradable carbohydrates, proteins, and lipids, which reduce the severity of pretreatment required (Pagliaccia et al. 2019). Its abundant availability, high moisture content, and rich organic fraction therefore make food waste an attractive substrate for sustainable bioethanol production, while simultaneously addressing the global challenge of improper waste disposal (Kazemi Shariat Panahi et al. 2022).

Food waste also contains a diverse array of nutrients—including carbohydrates, proteins, lipids, macro‐elements (N, P, K), and bioactive components—that make it suitable for biotechnological valorization (Ghorai et al. 2025). These constituents can be converted into a wide range of value‐added bioproducts, such as enzymes, bioethanol, biodiesel, biogas, biohydrogen, bioplastics, organic acids, biosurfactants, and pigments (Liu et al. 2023; Kumari et al. 2024). Among these, bioethanol is particularly relevant to this review because its production is strongly influenced by the effectiveness of pretreatment and hydrolysis steps, which determine fermentable‐sugar availability (Alvira et al. 2010; Tse et al. 2021). Understanding the physicochemical properties of food waste and their implications for pretreatment and fermentation is therefore fundamental to developing efficient and sustainable conversion pathways. Although food waste can be converted into several types of bioenergy—including biogas, biohydrogen, biodiesel, and biocoal—bioethanol is emphasized in this review because it offers distinct environmental and technological advantages.

Pretreatment is a crucial step in converting food waste biomass into fermentable substrates. Pretreatment methods—including physical, chemical, physicochemical, and biological approaches—aim to disrupt complex structures, increase surface area, reduce crystallinity, and improve enzymatic accessibility (Ravindran and Jaiswal 2016). Following pretreatment, enzymatic hydrolysis breaks down carbohydrates into simple sugars, which are subsequently transformed into bioethanol through microbial fermentation, the defining and yield‐determining stage of the entire process (Koppram et al. 2014). Multiple bioprocessing configurations—including SHF, SSF, SSCF, and consolidated bioprocessing—have been developed to integrate pretreatment, saccharification, and fermentation with varying degrees of efficiency and sustainability.

To compile this review, a targeted literature search was conducted using Scopus, Web of Science, ScienceDirect, and Google Scholar. Keywords such as “food waste”, “pretreatment”, “enzymatic hydrolysis”, “fermentation”, and “bioethanol” were used in various combinations. Studies published mainly between 2010 and 2024 were reviewed, with earlier foundational sources included when relevant. Articles were selected based on their relevance to food‐waste composition, pretreatment strategies, hydrolysis efficiency, and fermentation performance, while studies focused solely on non‐food biomass or non‐fermentative pathways were excluded.

Overall, this review examines the current status of food‐waste generation, its environmental implications, the physicochemical characteristics of food waste, and the pretreatment and hydrolysis strategies that enable its efficient conversion into bioethanol. By focusing on the integration of sustainable pretreatment techniques and optimized fermentation processes, the review highlights pathways to advance both renewable‐energy production and improved waste‐management practices within a circular‐economy framework.

## 2 Food Waste: Current Status and Global Dilemma

**FIGURE 1:**
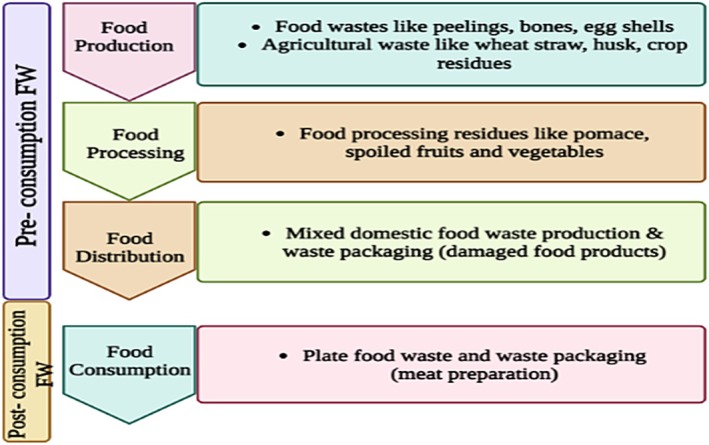
Difference between pre‐ and post‐consumption food waste.

Globally, food waste has emerged as a critical environmental and socio‐economic challenge. According to the Food and Agriculture Organization (FAO), nearly 1.3 billion tons of food—approximately one‐third of all food produced for human consumption—is wasted every year (McGuire 2015; Mc Carthy et al. 2018). This level of food wastage contributes significantly to greenhouse‐gas emissions, resource depletion, and financial losses estimated at US $936 billion annually. Projections further indicate that global food waste may exceed 2.2 billion tons by 2025, driven by population growth, urbanization, and changing consumption behavior (Mahmudul et al. 2022). Figure 1 illustrates distinctions between pre‐consumption and post‐consumption food waste.

In the regional context, India represents one of the largest contributors to food waste in Asia due to its enormous population, extensive agricultural output, and rapidly expanding urban food systems. India generates an estimated 68–70 million tons of food waste annually, arising from households, food‐service establishments, markets, and supply‐chain inefficiencies. Beyond food waste, India also generates large volumes of solid waste (1–3 billion tons annually across all categories), which further strains existing waste‐management infrastructure (Sahoo et al. 2023). The considerable fraction of this waste that is organic intensifies environmental concerns, especially due to methane emissions from landfilling.

The proliferation of food waste presents a growing concern globally, with projections indicating that by 2025, the worldwide production of such waste is poised to exceed 2.2 billion tons, driven by factors like economic growth and population expansion. Conventional methods for managing food waste, such as landfilling and incineration, pose substantial risks to both human health and the environment, chiefly due to the emission of harmful greenhouse gases (Ma and Liu 2019; Esparza et al. 2020; Zhu et al. 2023). The primary harmful gas emitted from landfilled food waste is methane (CH_4_), a potent greenhouse gas with a global warming potential approximately 28–34 times higher than carbon dioxide over a 100‐year period. In addition to methane, carbon dioxide (CO_2_) is also released during anaerobic decomposition, collectively intensifying the environmental burden associated with landfill disposal. Paradoxically, India—despite being the world's leading producer of milk—generates an estimated 1–3 billion tons of total solid waste annually, a figure that reflects overall waste generation across agricultural, industrial, and municipal sectors rather than milk‐derived waste. A considerable portion of this waste constitutes agricultural byproducts, exacerbating environmental pollution and public health concerns due to insufficient waste management practices (Table 1).

**TABLE 1:** Classification of food waste based on pre‐consumption and post‐consumption categories.

Category	Description	Examples
Pre‐consumption food waste	Waste generated before food is served or consumed, usually during preparation, storage, or processing. Often includes inedible parts removed prior to cooking.	Fruit and vegetable peelings; trimming residues; stalks; seeds; eggshells; fish scales; shells; animal bones; bakery trimmings; rejected produce; expired but unopened packaged foods; dairy/market sorting residues.
Post‐consumption food waste	Waste generated after food has been served to consumers, typically consisting of plate leftovers or uneaten cooked food.	Plate scrapings; half‐eaten meals; leftover cooked rice/pasta; uneaten bread; spoiled refrigerated meals; beverage residues; unfinished restaurant portions; household cooked food waste.

**FIGURE 2:**
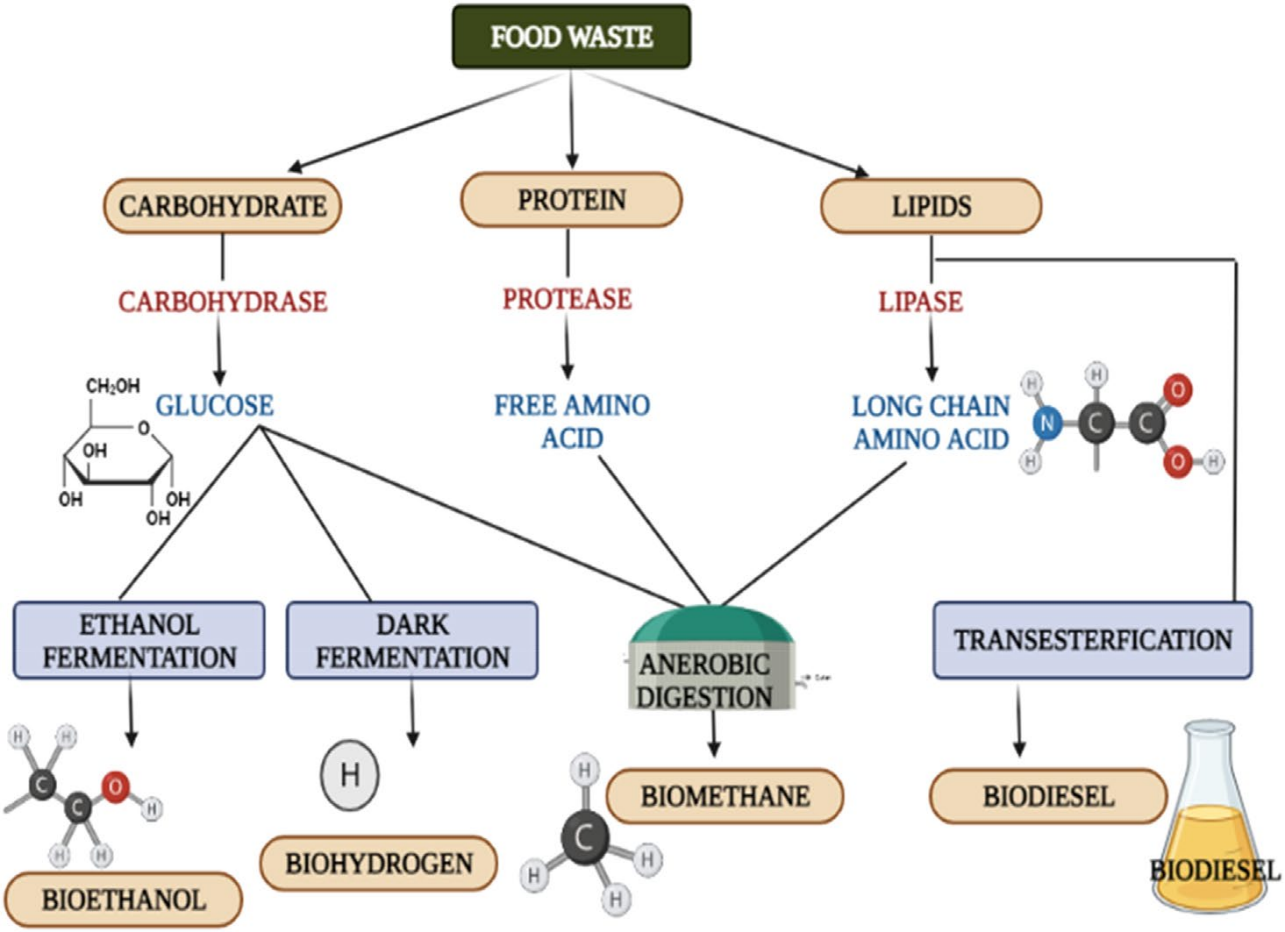
Various bio‐energies recovered from three organic components of food waste.

On a global scale, an astonishing 200 billion tons of agricultural lignocellulosic waste are generated annually, with India consistently contributing approximately 200 million tons to this substantial figure (Nanda et al. 2015; Prasad et al. 2019). Food waste consists primarily of three organic components that can be biologically converted into various forms of bioenergy, including biomethane, biohydrogen, bioethanol, and biodiesel (Dhanya et al. 2020; Sharma et al. 2024). The specific type of bioenergy produced is closely correlated with the organic components employed, as delineated in Figure 2.

Fruit and vegetable waste is produced at the end‐user level as a result of elements including refrigeration, heat treatment, and varied levels of ripeness. Waste generation is also influenced by several metabolic processes including phenolic chemicals, enzymes, antioxidants, and oxygen exposure. These reactions frequently lead to discoloration and ultimately microbial degradation, which causes problems like surface growth, rotting, and weakening (Rawat 2015; Bautista‐Zamudio et al. 2023). Another source of food waste is kitchen waste, which is produced during the process of preparing food. Inadequate storage, transportation, handling, and processing facilities are frequently the cause of leftovers.

Understanding global food‐waste generation is essential not only from a waste‐management perspective but also for identifying sustainable feedstocks for bioethanol production. Since food waste is an abundant second‐generation substrate with no competition for arable land, utilizing it for bioethanol contributes to both environmental sustainability and circular bioeconomy goals.

## 3 Properties of Food Waste

The properties of food waste can be categorized into two broad groups: physical properties and chemical properties. These characteristics are primarily influenced by the source of the food waste, its overall composition, and the specific constituents it contains. These properties determine how efficiently food waste can be pretreated, hydrolyzed, and fermented, which directly affects bioethanol yield and the overall sustainability of the conversion process.

These physicochemical characteristics directly influence the efficiency of pretreatment, enzymatic hydrolysis, and fermentation steps in bioethanol production. Therefore, understanding these properties is central to designing sustainable and efficient bioprocesses that maximize bioethanol yield while minimizing resource and energy consumption.

### 3.1 Physical Properties

Physical properties of food waste—such as particle size, bulk density, moisture content, and porosity—play a direct role in determining its suitability and efficiency in bioethanol production. Smaller particle sizes increase surface area and improve the effectiveness of pretreatment, allowing enzymes to access carbohydrates more easily during hydrolysis (Razzaghi et al. 2021). Bulk density influences the flow behavior and mixing efficiency in pretreatment reactors, affecting heat transfer and enzymatic penetration. Moisture content is also critical, as high moisture levels facilitate enzymatic hydrolysis and reduce the need for dilution prior to fermentation (Chew et al. 2018). Inadequate physical properties can hinder enzyme–substrate interactions, reduce fermentable‐sugar release, and ultimately lower bioethanol yields (Slopiecka et al. 2022). Therefore, understanding and optimizing the physical characteristics of food waste is essential for achieving efficient saccharification and robust fermentation performance.

#### 3.1.1 Bulk Density

The bulk density (BD) of food waste can be expressed as mass per unit volume and the unit BD can be expressed as kilograms per cubic meter (kg/m^3^). BD is a crucial parameter that reflects the porosity and compaction of waste materials. In the case of food waste, the bulk density typically falls within the range of 120–480 kg/m^3^. High BD values indicate low porosity and high density in food waste (Chew et al. 2018). A study compared the moisture and bulk density thresholds of food waste to determine the variation in bulk‐density‐based recognition methods. The research aimed to understand the dominant controlling subcomponents in food waste and their relationship with bulk density (Li, Zhang, et al. 2022). Another study presented a simple recognition method for kitchen and dry waste based on bulk density. The research aimed to improve waste management practices by accurately identifying and categorizing different types of waste (Li et al. 2020). A study focused on the physical and chemical characteristics of food waste, including density, composition, generation rate, pH, and moisture content. The research aimed to develop effective strategies for managing food waste in a university setting (Zhan et al. 2017; Waqas et al. 2018). Composting food waste offers a chance to reduce emissions, but there is a paucity of information on the entire pile, commercial‐scale emissions, and the underlying biogeochemical processes. This study examined the potential of composting food waste to mitigate climate change and included the measurement of bulk density as a criterion (Nweze et al. 2023). The quality of the wastes investigated in this study, with low pH, high organic acid content, and lactic acid bacteria present, poses a serious challenge for composting processes. Bulk density measurements can help in understanding the composition and behavior of the waste during composting (Feng and Zhang 2022). For bioethanol production, appropriate bulk density improves feeding consistency in pretreatment reactors and enhances mass‐transfer efficiency, which ultimately supports more uniform hydrolysis and higher fermentable‐sugar recovery.

#### 3.1.2 Moisture Content

Moisture content in food waste refers to the quantity of water present in food waste or the amount of water that evaporates when food waste is dried at a consistent temperature of 105°C (Liu et al. 2020; Lawrance et al. 2022). Typically, the moisture content of food waste falls within the range of 50%–80% of its dry weight, meaning that a significant portion of its weight consists of water. It's worth noting that as the moisture content of food waste increases, its actual weight also increases. Some researchers have observed that higher water content in food waste encourages the formation and expansion of particle aggregates (Fausto‐Castro et al. 2020; Bandini et al. 2020). However, it's important to note that increasing the water content in food waste tends to reduce its porosity and lower its pH. Moisture content strongly influences pretreatment energy demand and enzymatic hydrolysis efficiency. High moisture levels facilitate enzymatic activity, reducing the need for dilution and supporting a more sustainable conversion of food waste into bioethanol.

Optimizing these physical characteristics enhances the sustainability of bioethanol production by improving hydrolysis performance and reducing the energy input required for pretreatment.

### 3.2 Chemical Properties

Food waste typically contains a heterogeneous mixture of carbohydrates, lipids, proteins, fibers, minerals, and moisture, with the exact composition depending on the source (household, market, restaurant, or industrial food processing). Among these components, carbohydrates—particularly starches, sugars, and cellulose—are the most critical for bioethanol production, as they serve as the primary substrate for enzymatic hydrolysis and microbial fermentation. High carbohydrate content generally correlates with greater fermentable‐sugar availability and higher potential bioethanol yields. Proteins and lipids, although not directly fermentable into ethanol, influence process performance by affecting pH, nitrogen availability, and the formation of inhibitory compounds during pretreatment. The presence of fibers and structural polysaccharides (e.g., hemicellulose and lignin) determines the severity of pretreatment required to increase enzyme accessibility (Yukesh Kannah et al. 2020; Sarangi et al. 2023). Understanding this compositional profile is essential for selecting suitable pretreatment strategies and optimizing hydrolysis and fermentation steps in the bioethanol production pathway. Food waste is highly heterogeneous, but its approximate biochemical composition generally falls within identifiable ranges. On a dry‐weight basis, food waste typically contains carbohydrates (30%–60%), proteins (5%–20%), lipids (10%–40%), moisture (50%–80% of fresh weight), and ash (2%–10%) (Cheng et al. 2017; Ho and Chu 2019). These values vary depending on the type of waste—fruits and vegetables are richer in carbohydrates and moisture, whereas kitchen and restaurant wastes tend to have higher lipid and protein fractions. Providing these approximate ranges helps contextualize the influence of composition on pretreatment selection, enzymatic hydrolysis efficiency, and the fermentable‐sugar yield essential for bioethanol production.

#### 3.2.1 pH

Chemical characteristics such as pH are crucial to the bioconversion of food waste. The activity of the microorganisms participating in the bioconversion process is influenced by the pH of the food waste, which has an impact on the efficiency and effectiveness of the process. The optimal pH range for anaerobic digestion, a common bioconversion process, is between 6.5 and 8.5 (Chew et al. 2021; Aworanti et al. 2023). If the pH is too low or too high, the activity of microorganisms can be inhibited, leading to a decrease in the efficiency of the process. For example, a low pH can lead to the accumulation of volatile fatty acids, which can inhibit the activity of methanogens, while a high pH can lead to the accumulation of ammonia, which can be toxic to microorganisms (Czatzkowska et al. 2020). Therefore, maintaining the optimal pH range is crucial for the success of the bioconversion process. The pH of food waste can vary depending on the type of waste and its composition. For example, the pH of food waste used in a study ranged from 6.67 to 6.82 (Islam et al. 2023), while another study reported a pH value of 7.29 for compost made from agricultural waste (Al‐Suhaibani et al. 2020). The primary factor in food waste that enhances microbial activity during various bioconversion processes is its pH level. The pH value is a measure of the concentration of hydrogen ions in food waste and typically falls within the range of 4.2–5.3, which can vary depending on the type and quantity of organic materials present (Yukesh Kannah et al. 2020). Food waste is often characterized by its acidic nature. In the context of various bioconversion processes, microbial activity is most successful when the pH levels are close to neutral or slightly acidic. For instance, in hydrogen production processes, the optimal pH range typically falls between 5 and 5.5, while for methane production, it is within the range of 6.9–7.3 (Aashabharathi et al. 2024). Maintaining a suitable pH is essential for both hydrolytic enzymes and fermenting microorganisms, making it a critical factor in achieving efficient and stable bioethanol fermentation from food waste.

#### 3.2.2 Electrical Conductivity

Another crucial chemical characteristic that is crucial to the bioconversion of food waste is electrical conductivity (EC). It is frequently used to describe a material's capacity for transferring electrons, with higher conductivity implying that more electrons are moved in a given amount of time (Voběrková et al. 2020). In the context of food waste bioconversion, electrical conductivity can influence the efficiency and effectiveness of the process in the following ways: Higher electrical conductivity can indicate the presence of conductive materials that facilitate direct interspecies electron transfer (DIET) in anaerobic digestion. This can alleviate the inhibition of microorganisms and enhance methanogenesis, leading to improved bioconversion efficiency (Li et al. 2021). Sludge's electrical conductivity can be utilized as a feature to assess how anaerobic digestion systems can improve their ability to transmit electrons. This can assist academics and professionals in evaluating the efficiency of various bioconversion techniques and materials (Wu et al. 2023). The addition of conductive materials, such as biochar prepared at specific pyrolysis temperatures, can improve the electrical conductivity of sludge. This, in turn, can enhance the efficiency of the bioconversion process by facilitating electron transfer and promoting the activity of microorganisms (di Chen et al. 2020; Yu et al. 2022). Changes in electrical conductivity during the bioconversion process can be used to monitor the performance and stability of the system. For example, the continuous strengthening of sludge conductivity with the addition of conductive materials can indicate the positive effects of these materials on the bioconversion process (Wu et al. 2022). The property of food waste that permits electric current to flow through it is referred to as its EC and is commonly expressed in units of dS/m. The primary element facilitating the flow of electric current in food waste is the presence of ionic compounds in the aqueous solution. In the aqueous phase, there is a significant concentration of ionic components, which results in increased electrical conductivity. Simply said, the concentration of total dissolved solids or the salinity level has a direct impact on the EC of food waste. The relationship between EC and salinity or total dissolved solids concentration is direct. In the context of bioethanol production, electrical conductivity can influence microbial metabolism and fermentation stability, thereby affecting overall conversion efficiency.

#### 3.2.3 Proximate Analysis

A crucial role in the bioconversion of food waste is played by the chemical property proximate analysis. Proximate analysis reveals the material's waste nature by providing data on the amount of moisture, total solids, and volatile solids in organic components (Tumuluru et al. 2021). The composition of food waste can be ascertained through proximate analysis, which is crucial for planning and improving bioconversion processes. According to the source and kind of waste, the composition of food waste can vary, and proximate analysis can assist in determining the ideal circumstances for bioconversion. For instance, research that examined the makeup of restaurant food waste reported the following composition: Crude fat makes up 31.8% of the whole solid, followed by cellulose, hemicellulose, lignin, crude protein, 15.5%, and carbohydrates, 41.6% (Muthu et al. 2023). This information can be used to optimize the bioconversion process by adjusting the operating conditions to maximize the conversion of specific components. Additionally, proximate analysis can be used to monitor the progress of the bioconversion process by measuring changes in the composition of food waste over time (Ortigueira et al. 2019). This can help researchers and practitioners assess the effectiveness of different bioconversion strategies and materials. The actual weight of the waste increases with higher moisture and ash content, but this doesn't impact the heating value of the food waste (Chhandama et al. 2022). Volatile matter is the term used to describe the portion of trash that burns to produce petrol or liquid fuel. On the other hand, fixed carbon is the small quantity of residue that is still there after all of the trash has been completely burned, and it resembles charcoal in certain ways (Güler and Aydın Temel 2023). Proximate analysis helps determine the amount of carbohydrates, proteins, and lipids available, which is essential for estimating the theoretical bioethanol potential of food waste and optimizing pretreatment severity.

#### 3.2.4 Ultimate Analysis

The ultimate analysis, also referred to as elementary analysis, is a method employed to quantify the presence of the five primary elements in a mixture, which are carbon (C), hydrogen (H), nitrogen (N), oxygen (O), and sulfur (Muthu et al. 2023). The composition of food waste can be determined using the data from the final analysis, which is crucial for designing and refining the bioconversion processes. Depending on the source and type of waste, the content of food waste might vary, and a final examination can assist in determining the ideal circumstances for bioconversion. For instance, the final examination of food waste can reveal the amount of carbon and nitrogen present, allowing the C/N ratio to be adjusted to best suit the bioconversion process (Workie et al. 2023). Additionally, ultimate analysis can be used to monitor the progress of the bioconversion process by measuring changes in the composition of food waste over time. This can help researchers and practitioners assess the effectiveness of different bioconversion strategies and materials. Ultimate analysis, particularly the C/N ratio, directly affects microbial growth and fermentation performance, making it an important indicator of bioethanol production feasibility.

#### 3.2.5 Heavy Metals Content

Heavy metal content is a chemical property that can have a significant impact on food waste bioconversion. Heavy metals are toxic to microorganisms and can inhibit the activity of enzymes involved in the bioconversion process. Arsenic (As), cadmium (Cd), mercury (Hg), chromium (Cr), copper (Cu), thallium (Tl), iron (Fe), nickel (Ni), magnesium (Mg), manganese (Mn), lead (Pb), and zinc (Zn) are only a few of the heavy metals that may be present in traces in food waste (Verma and Kuila 2019; Diaconu et al. 2020; Sharma and Kumar 2021). If not handled and disposed of appropriately, these heavy metals have the potential to harm the ecosystem. Several variables, such as the origins of the food waste and the potential for contamination during food processing, might affect the presence of heavy metals in food waste (Chu et al. 2019; O'Connor et al. 2021). The ecosystem and human health may both be in danger from the presence of heavy metals in food waste. As a result, it's critical to check that the heavy metal level of food waste is within acceptable bounds. The activity of microorganisms participating in the bioconversion process can be inhibited by heavy metals, which lowers the process' efficiency. For instance, it has been demonstrated that the presence of lead and cadmium in food waste inhibits the action of methanogens during anaerobic digestion (Luo et al. 2020). The use of compost made from food waste with high heavy metal content can be toxic to plants and reduce crop yields. Therefore, it is important to ensure that the heavy metal content of food waste is within safe limits before using it as a fertilizer (Guo et al. 2020). Heavy metals can also pose a risk to the environment if they are not properly disposed of. For example, heavy metals can leach into groundwater and contaminate soil, leading to long‐term environmental damage (Mukherjee et al. 2021). Heavy metal content is an important chemical property that needs to be monitored to ensure the safe and effective bioconversion of food waste. For bioethanol production, excessive heavy metals can inhibit both hydrolytic enzymes and fermenting microorganisms, reducing conversion efficiency. Assessing metal content is therefore crucial for ensuring stable and sustainable bioethanol fermentation.

Heavy metals present in food waste can originate from multiple sources:

- Agricultural inputs, such as fertilizers, pesticides, and contaminated irrigation water, which may introduce metals like cadmium (Cd), lead (Pb), and arsenic (As) into crops (Alengebawy et al. 2021; Khatun et al. 2022).
- Food processing and handling equipment, including metal grinders, mixers, and storage containers, which may contribute iron (Fe), copper (Cu), or chromium (Cr) residues (Daniel et al. 2021).
- Packaging materials, especially inks, coatings, and metallic foils, which can leach trace metals into discarded food items (Deshwal and Panjagari 2019).
- Environmental contamination during transport, storage, or preparation, particularly in urban areas with high air or dust pollution (Vlasov et al. 2022).

These metals are important to monitor because they can inhibit enzymatic hydrolysis and fermentation, thereby reducing bioethanol yield and affecting process stability.

#### 3.2.6 Biopolymer Content

Food waste primarily consists of main biopolymers: carbohydrates (30%–60%) and proteins (5%–20%) (Sharmila et al. 2020). The composition and proportions of these biopolymers can vary based on the specific components present in the food waste. For example, if food waste contains a significant amount of rice and vegetables, the concentration of carbohydrates tends to increase. On the other hand, if food waste includes meat and eggs, the concentrations of proteins and lipids become more prominent (Sahu et al. 2024). Fruit and vegetable waste normally has a low lipid concentration, whereas kitchen trash might have a high lipid level. It has been found that the overall lipid content in fruits and vegetables is about 11.8% of the total, while the total lipid content in kitchen trash is about 21.6% (Bong et al. 2018). This lipid content can be advantageous for increasing the production of liquid biofuels, specifically biodiesel. The relative proportions of carbohydrates, proteins, and lipids determine the number of fermentable sugars released during hydrolysis, making biopolymer composition a key predictor of bioethanol yield from food waste.

These chemical parameters govern microbial activity and enzyme performance, ultimately determining the feasibility and sustainability of converting food waste into bioethanol.

These physicochemical characteristics not only influence pretreatment and hydrolysis efficiency but also affect the downstream fermentation stage, where fermentable sugars are biologically converted into bioethanol.

## 4 Pretreatment of Food Waste Biomass

**FIGURE 3:**
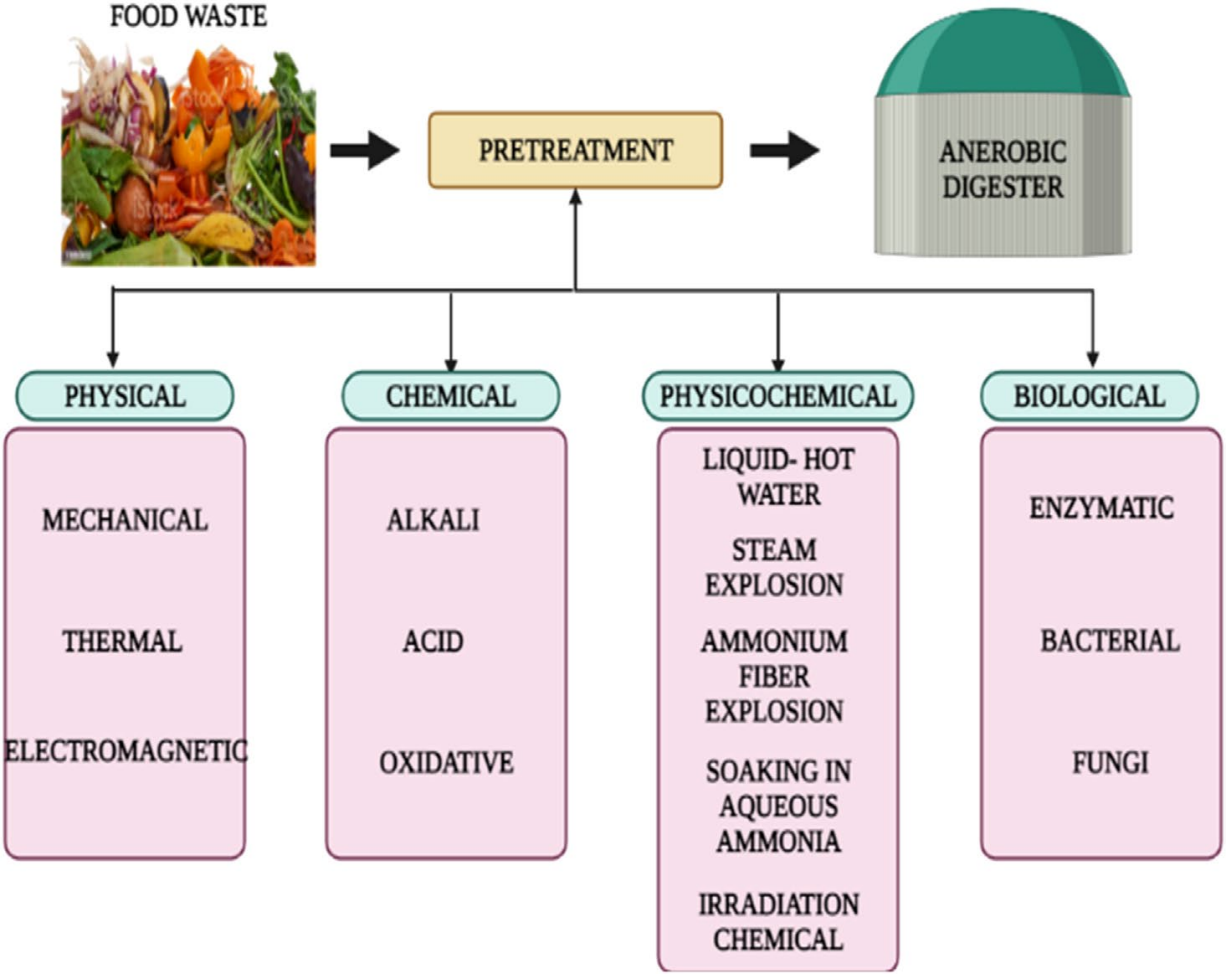
Different types of food waste pretreatment techniques.

Pretreatment is a critical step in converting food waste into bioethanol because it breaks down complex structural components—such as starches, fibers, hemicellulose, and lignin—thereby increasing the accessibility of carbohydrates to hydrolytic enzymes (Hernández‐Beltrán et al. 2019; Tan et al. 2021). Effective pretreatment enhances the release of fermentable sugars, reduces crystallinity, disrupts structural integrity, and improves the efficiency of subsequent enzymatic hydrolysis and microbial fermentation (Li, Zhu, et al. 2019; Kainthola et al. 2019). Unlike pretreatment strategies designed for anaerobic digestion, which focus primarily on increasing biodegradability for methane production, pretreatments for bioethanol production emphasize maximizing sugar availability and minimizing the formation of inhibitors that can affect fermentation. Figure 3 shows the different types of pretreatments for different food waste. Since pretreatment strongly influences fermentable‐sugar release, it directly affects bioethanol yields and thus plays a critical role in advancing sustainable waste‐to‐fuel pathways. Ultimately, the effectiveness of any pretreatment method is evaluated by its ability to improve fermentability, as fermentation is the step where sugars are transformed into bioethanol.

Pretreatment is critical for maximizing the biodegradability of food waste before aerobic or anaerobic digestion. Complex organic matter is solubilized and transported to the aqueous phase during this process, making it easier for enzymes to access and speeding up the overall breakdown process (Fisgativa et al. 2018; Li, Zhu, et al. 2019). Pretreatment often serves to increase the surface area of food waste, making it more conducive to efficient biodegradation. Additionally, it reduces the degree of polymerization and crystallinity in food waste, which in turn accelerates the rate of hydrolysis. Table 2 tabulates the characteristics, merits, and demerits of physical, chemical, and biological pretreatment processes. Before aerobic digestion, pretreatment is typically employed to effectively reduce substrate particle size, increase the number of soluble organics in the aqueous phase, and minimize the concentration of inert compounds that obstruct digestion. There are four main pretreatment techniques: physical (thermal, microwave), chemical (acid, alkaline, ozone, surfactant), biological (enzyme, microorganisms), and physicochemical (combination of physical and chemical processes) (Yukesh Kannah et al. 2020; Uthirakrishnan et al. 2022; Awogbemi and Von Kallon 2022).

**TABLE 2:** Characteristics, merits, and demerits of different treatment processes.

Treatments	Characteristics	Merits	Demerits	References
Physical	Hydrolysis of cellulose, lignin, and hemicellulose. De‐crystallization of waste structure. Depolymerization of cellulose, lignin, and hemicellulose. Milling, steam explosion, pressure treatments, irradiation, and other mechanical and thermal treatments.	Time efficient. Environment friendly	Expensive Huge energy requirements	Hassan et al. (2018), Jȩdrzejczyk et al. (2019), Mankar et al. (2021), and Singh et al. (2023)
Chemical	Effective for lignin breakdown Alkali or acid hydrolysis, organic solvents, ozone exposure.	Specific Quicker reaction time Effective for breaking tough biomass	Expensive Hazardous Non‐selective reaction	Kumari and Singh (2018), Hassan et al. (2018), Mankar et al. (2021), Norrrahim et al. (2021), and Sai Bharadwaj et al. (2023)
Enzymatic	Selective degradation of cellulose, hemicellulose, and lignin via catalytic action of lignocellulolytic enzymes	Specific Low energy requirements Eco‐ friendly	Time‐consuming Slow reaction rate Expensive	Hosseini Koupaie et al. (2019), Sheng et al. (2021), Li, Shi, et al. (2022), and Zhou, Li, et al. (2023)

### 4.1 Physical Pretreatment

A widely used technique for increasing the effectiveness of composting and biogas production is physical pretreatment. Physical pretreatment relies on mechanical, thermal, or electromagnetic methods to alter lignocellulosic structure, reduce crystallinity, and increase porosity (Hassan et al. 2018; Taylor et al. 2019). Physical pretreatment techniques do not require the addition of additional materials, such as chemicals or microorganisms, like chemical and biological pretreatment techniques do. However, it's crucial to keep in mind that physical pretreatment procedures tend to generate a sizable amount of refractory chemicals and are energy‐intensive, requiring the use of power and heat (Kumari and Singh 2018). Mechanical pretreatment involves the reduction of substrate particle size using various equipment such as shredders and mills. These can include hammer mills, knife mills, bead mills, wet disc mills, and others. Typically, these processes result in substrates with diameters of less than 2 mm (for milling) or between 10 and 30 mm (for chopping). These techniques are generally suitable for substrates with moisture content less than 15%. For substrates with higher moisture content, typically greater than 15%–20% on a wet basis, colloid mills, extruders, and expanders are more appropriate for achieving effective mechanical pretreatment (Rusanowska et al. 2018; Dell'Omo and Spena 2020). Thermal pretreatment includes heating food waste, which causes cell membranes to fall apart and organic components to become soluble. It's crucial to remember that thermal pretreatment at extremely high temperatures—typically greater than 170°C—can harm anaerobic digestion. Complex substrates that are difficult to degrade may form at such high temperatures, which could reduce the anaerobic digestion process' effectiveness (Hassan et al. 2018; Singh et al. 2023).

Utilizing physical pretreatment methods to enhance biogas production and composting efficiency is lauded for its eco‐friendly approach, as it eliminates the need for additional substances like chemicals or microorganisms, simplifying processes and reducing potential hazards. The focus on modifying lignocellulosic materials, by reducing crystallinity and increasing surface area, shows a keen understanding of structural influences on bioconversion. However, energy intensity poses a drawback, increasing operational costs and environmental impacts. Careful assessment of energy demand and associated emissions is necessary for overall sustainability. Managing recalcitrant compounds and ensuring precise temperature control during thermal pretreatment is crucial for maintaining anaerobic digestion efficiency. Solutions include optimizing energy use, employing renewable sources, managing recalcitrant compounds, and implementing precise temperature monitoring. A thorough life cycle assessment can guide decision‐making and identify areas for improvement. Balancing the benefits with challenges is crucial for sustainable implementation in food waste management practices. This pretreatment enhances fermentable‐sugar release and improves compatibility with downstream enzymatic hydrolysis and fermentation for bioethanol production.

### 4.2 Chemical Pretreatment

Chemical pretreatment entails applying chemicals to change the amorphous form of food waste or biomass so that enzymes can access it more easily. The procedure can use a variety of alkalis and acids at varying concentrations, with the biomass or food waste being submerged in the chemical solution and, in some situations, heated for a predetermined amount of time (Hoang et al. 2021). This treatment causes the pores of the material to swell, thereby increasing its surface area. However, it is important to note that chemical pretreatment is not considered highly durable or environmentally friendly because it can release harmful chemicals into the environment (Zoghlami and Paës 2019; Okolie et al. 2021). Depending on the individual substrate, the efficacy of chemical pretreatments might vary greatly; therefore, it is critical to optimize the chemicals used, their doses, and the length of the treatments to get the best outcomes.

Acid pretreatment methods generally involve the use of either concentrated or diluted acid solutions. Concentrated acids typically require solutions with concentrations greater than 30% (w/v) and are conducted at temperatures below 100°C for several hours. However, one significant drawback of using concentrated acids is that they necessitate the use of corrosion‐resistant materials because higher concentrations are more corrosive (Galbe and Wallberg 2019; Hoang et al. 2021). Additionally, a neutralizing agent is often required to adjust and raise the pH level. In contrast, diluted acid pretreatment uses acid solutions with concentrations ranging from 0.5% to 5% (w/v) but involves elevated temperatures ranging from 120°C to 215°C and shorter treatment times, typically lasting for several minutes (Taherzadeh and Karimi 2008).

Alkaline chemicals, such as sodium hydroxide, lime, ammonia, and others, are frequently used to treat substrates and have been the subject of much research. This technique works well to break down lignin and improve microbial access to cellulose and hemicellulose (Pellera and Gidarakos 2018; Beig et al. 2021). To reduce the pH to acceptable levels following an alkaline pretreatment, though, a neutralizing substance like sulfuric acid might be required. While chemical approaches have several benefits, such as effective biomass fractionation and good sugar yields during enzymatic hydrolysis, they also have some environmental disadvantages. These negatives include the possibility of biomass degradation into chemicals that block enzymes, reactor corrosion, difficulties with solvent recycling, and trash production, all of which might have a greater environmental impact than physical and biological pretreatment techniques (Sołowski et al. 2020).

Chemical pretreatment is a valuable technique for improving the accessibility of food waste or biomass to enzymes, enhancing its suitability for bioconversion processes. By employing acids and alkalis, this method modifies the material's structure, increasing surface area and enzymatic digestibility. It effectively breaks down complex lignocellulosic structures, leading to higher sugar yields crucial for biofuel production. Despite its effectiveness, environmental concerns arise due to chemical usage and waste disposal. The generation of enzyme‐inhibiting compounds and reactor corrosion are additional challenges. To address these issues, prioritizing green chemistry principles, such as eco‐friendly solvents and waste minimization, is crucial. Efficient waste management strategies and research into alternative pretreatment methods are also essential for sustainable use. Maximizing the benefits of chemical pretreatment while minimizing its negative impacts requires a concerted effort to adopt environmentally friendly practices and continuous innovation in pretreatment techniques. This pretreatment enhances fermentable‐sugar release and improves compatibility with downstream enzymatic hydrolysis and fermentation for bioethanol production.

### 4.3 Enzymatic Pretreatment

Pretreatment serves to modify the structural complexity of food biomass, changing its size, shape, and composition. This alteration makes the biomass more amenable to hydrolysis by enzymes, ultimately breaking it down into sugars. Enhancing enzymatic accessibility to these structures is a crucial step, particularly because hydrolysis and subsequent processing of non‐pre‐treated biomasses tend to be slow, costly, labor‐intensive, yield‐poor, and time‐consuming (Li, Shi, et al. 2022). The most environmentally friendly approach to pretreatment involves biological treatment, where microbes and their enzymes play a central role in rendering food biomass highly usable for various purposes. This approach leads to a reduction in waste generation, environmental degradation, and energy consumption, making it a sustainable choice (Hosseini Koupaie et al. 2019).

Enzymatic pretreatment shows promise in modifying food biomass for various applications, offering eco‐friendly and sustainable solutions by utilizing microbial and enzymatic treatment. While enhancing accessibility for subsequent hydrolysis, challenges such as food waste composition variability and specific condition requirements persist. Lengthy enzymatic reactions and the need for tailored enzymes further complicate the process. To overcome these challenges, a thorough characterization of food waste composition is essential for optimizing enzymatic strategies. Research should focus on enzyme optimization and integration into larger bioprocesses for efficient bioenergy production. Robust monitoring and control systems can maintain optimal conditions. Addressing these challenges ensures enzymatic pretreatment's potential to convert food waste into valuable bioenergy and bioproducts, fostering sustainable and economically viable processes. This pretreatment enhances fermentable‐sugar release and improves compatibility with downstream enzymatic hydrolysis and fermentation for bioethanol production. Given its low energy demand, minimal chemical usage, and high specificity, enzymatic pretreatment aligns closely with the sustainability goals of bioethanol production, making it a key strategy in green biorefineries.

## 5 Enzymes Involved in the Degradation of Food Waste

**FIGURE 4:**
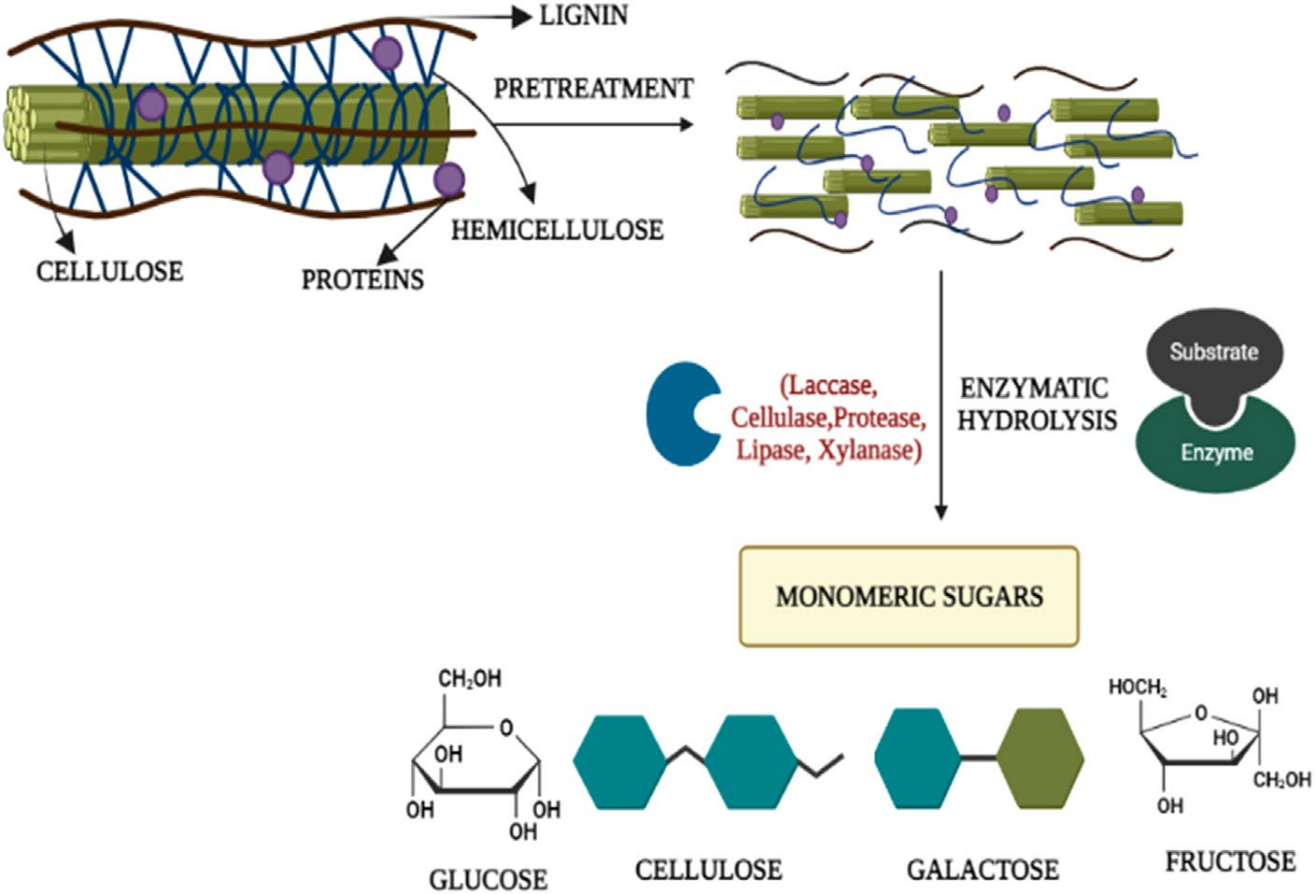
Schematic presentation of pretreatment effect and enzymatic hydrolysis on biomass.

Hydrolysis stands as the primary method for converting polymers into monomers and/or intermediates. Enzymatic hydrolysis plays a vital role in promoting the hydrolysis of food waste and reducing volatile suspended solids (Zou et al. 2020). Biological approaches typically emphasize the use of enzymes for the initial treatment of plant‐derived biomass. Enzymes are employed to decrease cellulose polymerization, break down hemicellulose through hydrolysis, and eliminate lignin from the biomass. Table 3 summarizes the vital roles played by enzymes in the degradation of food waste. The action of these enzymes enhances fermentable‐sugar availability, enabling efficient conversion of food waste into bioethanol and supporting sustainable valorization strategies. The release of simple sugars through enzymatic hydrolysis directly impacts the success of fermentation, underscoring the strong interdependence between enzymatic activity and bioethanol productivity as illustrated in Figure 4.

**TABLE 3:** Enzymes with their involvement in food waste degradation and their mechanism of action.

Enzyme	Involvement in food waste degradation	Mechanism of action	References
Cellulase	Breaks down cellulose, a major component of plant cell walls, into glucose and other sugars	Hydrolyzes the β‐1,4‐glycosidic bonds in cellulose to release individual sugar monomers.	Falarz et al. (2018), Houfani et al. (2020), Wang et al. (2020), and Zhou et al. (2021)
Hemicellulase	Degrades hemicellulose, a complex polysaccharide in plant cell walls, into simpler sugars	Catalyzes the hydrolysis of the various glycosidic bonds in hemicellulose, releasing xylose, glucose, mannose, and other sugars.	Houfani et al. (2020), Méndez‐Líter et al. (2021), de Souza and Kawaguti (2021), Qaseem et al. (2021), and Puchart et al. (2023)
Xylanase	Targets xylan, a type of hemicellulose, in plant cell walls, breaking it down into xylose and other oligosaccharides	Hydrolyzes the β‐1,4‐glycosidic bonds in xylan to release xylose and shorter oligosaccharides.	Kumar and Naraian (2019), Wang and Arioka (2021), Zerva et al. (2021), and Pandey et al. (2023)
Protease	Hydrolyzes proteins into amino acids, enabling the degradation of protein‐rich food waste components	Cleaves peptide bonds within proteins, breaking them down into individual amino acids.	Aspevik et al. (2017), López‐Pedrouso et al. (2019), and Zhou, Zhang, and Wang (2023)
Laccase	Oxidizes phenolic and non‐phenolic compounds, including lignin, in food and agricultural residues	Catalyzes the oxidation of phenolic and non‐phenolic substrates, leading to the breakdown of complex molecules, such as lignin.	Wang et al. (2018), Unuofin et al. (2019), Mayolo‐Deloisa et al. (2020), Singh and Gupta (2020), and Cagide and Castro‐Sowinski (2020)
Lipase	Degrades fats and oils into fatty acids and glycerol, facilitating the breakdown of lipid‐rich food waste	Hydrolyzes ester bonds in triglycerides, yielding glycerol and fatty acids. Lipases are essential in the conversion of fats into biodiesel.	Meghwanshi and Vashishtha (2018), Lopes et al. (2020), Mhetras et al. (2021), and Deaver and Popat (2022)

While enzymes can efficiently hydrolyze carbohydrates, proteins, and lipids in food waste, it is important to note that heavy metals cannot be degraded by enzymes, as they are elemental in nature. However, certain biological processes can alter their chemical form or reduce their toxicity. Microbial enzymes such as oxidoreductases, phosphatases, and metallothioneins can assist in the immobilization, complexation, or transformation of heavy metals, reducing their bioavailability (Olaniran et al. 2013; Rahman and Singh 2020; Ding et al. 2024). In cases where food waste contains elevated heavy‐metal concentrations, external pretreatments—including biosorption using microbial biomass, bioleaching by acid‐producing microorganisms, or enzymatic‐assisted immobilization—may be applied prior to hydrolysis and fermentation to prevent inhibition of fermentative microorganisms. Several reviews highlight these biological detoxification strategies for heavy‐metal‐contaminated wastes (Wang et al. 2021; Maqsood et al. 2022; Zhou, Zhang, and Wang 2023), emphasizing their relevance for improving the safety and efficiency of bioethanol production from food waste.

## 6 Production of Bioethanol From Food Waste

**FIGURE 5:**
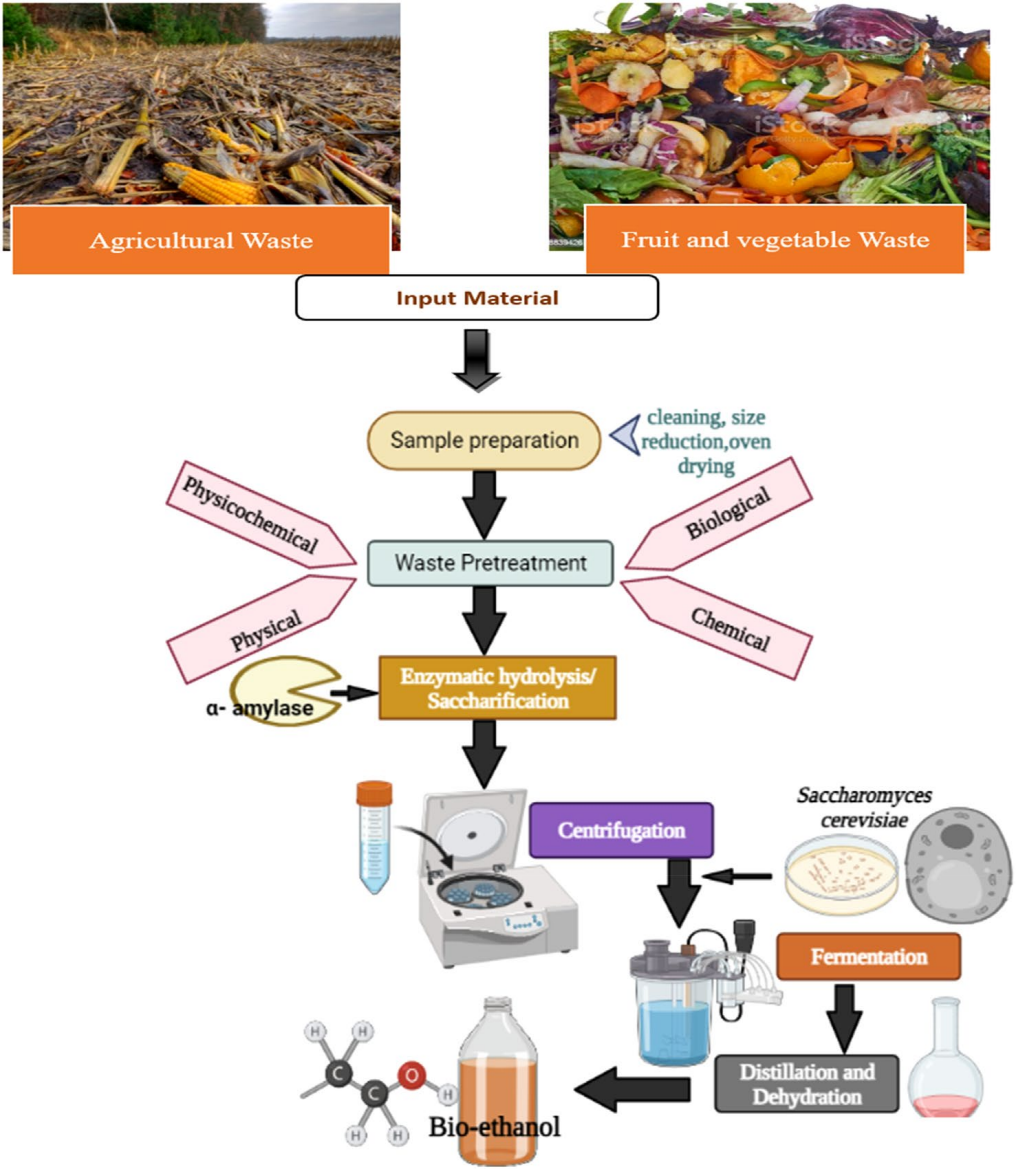
Schematic presentation of the process of bioethanol production.

Fermentation represents the core of the bioethanol production process, as it is the stage in which microorganisms convert hydrolyzed sugars into ethanol. The overall efficiency of bioethanol production therefore relies heavily on achieving optimal fermentation conditions and selecting robust fermentative strains. Recent years have seen a lot of interest in the use of food waste as a substrate for biofuel production, which not only resolves the issue of solid waste but also produces alternative energy sources (Ambaye et al. 2021). Starch, protein, and fat are the key components of food waste, and they make excellent carbon sources for fermentative biofuel production (Srivastava et al. 2021; Zeng et al. 2022). Food waste primarily consists of carbohydrates, which can be hydrolyzed into monomers and utilized as feedstock to produce biofuel (bioethanol). As shown in Figure 5, pretreatment, hydrolysis/saccharification, fermentation, and distillation are the steps in the production of bioethanol. Producing bioethanol from food waste aligns with sustainable development principles by diverting organic residues from landfills, reducing methane emissions, and generating renewable low‐carbon fuel.

A critical examination of different food‐waste feedstocks shows that those with high carbohydrate content, low lignin fractions, and minimal pretreatment requirements consistently yield the best results for bioethanol production. Fruit and vegetable wastes are advantageous due to their high moisture and simple sugar content, enabling rapid fermentation with minimal pretreatment (Cabas Candama et al. 2020). Similarly, starchy wastes (e.g., cooked rice, potatoes, pasta, bakery waste) contain readily hydrolysable polysaccharides that support high fermentable sugar release and elevated bioethanol yields (Onyeaka et al. 2022). In contrast, mixed kitchen waste and meat‐rich residues require more intensive pretreatment due to higher lipid and protein fractions, which may generate inhibitory compounds. Based on current literature, starchy and sugary food wastes are considered the most efficient and sustainable feedstocks for bioethanol production, offering both high conversion efficiency and low energy input during pretreatment.

### 6.1 Bioprocessing Strategies Used for the Bioconversion of Food Waste Into Bioethanol

All bioprocessing strategies for bioethanol production—SHF, SSF, SSCF, and CBP—are designed around enhancing fermentation efficiency, highlighting its pivotal role in the conversion of food waste into renewable fuel. Bioprocessing strategies using microorganisms and enzymes for bioethanol fermentation include separate hydrolysis and fermentation, simultaneous saccharification and fermentation (SSF), simultaneous saccharification and co‐fermentation (SSCF), simultaneous saccharification, filtration, and fermentation, and integrated/consolidated bioprocessing (CBP) as some of the methods used to ferment monosaccharides into bioethanol. Batch, fed‐batch, continuous, and solid‐state fermentation are additional forms of fermentation. Below is a description of several of these fermentation processes (Edeh et al. 2020). These bioprocessing strategies must therefore be evaluated not only for technical performance but also for their contribution to sustainable and circular bioethanol production systems.

#### 6.1.1 Separate Hydrolysis and Fermentation (SHF)

**FIGURE 6:**
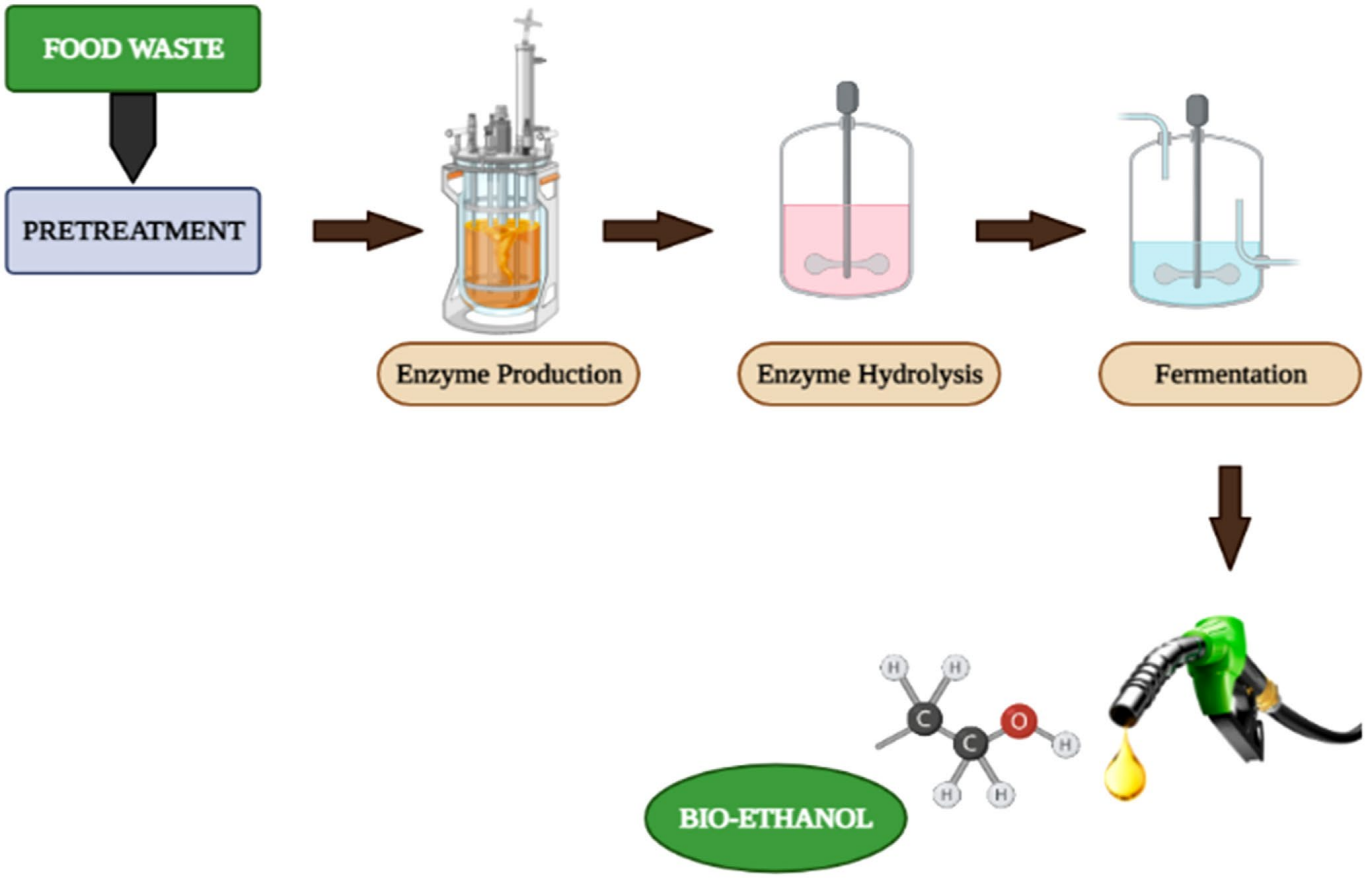
Separate hydrolysis/saccharification and fermentation for bioethanol production.

As seen in Figure 6, this technique is typically employed to convert carbohydrates into bioethanol. Additionally, it offers ideal conditions for the fermenting organisms and enzymes or other catalysts utilized in the fermentation and saccharification processes, respectively. Because hydrolytic enzymes and fermenting organisms' function at their best, high bioethanol yield is anticipated. In SHF, hydrolysis is completed before microorganisms are added for fermentation, and sugars may be recovered through centrifugation or filtration (Singh et al. 2022). High capital expenditures, particularly as a result of the demand for two reactors, lengthy reaction times, and end product inhibition—where the cellobiose and glucose produced as a result of enzymatic hydrolysis restrict cellulose activity—are disadvantages of SHF (Reis et al. 2023).

#### 6.1.2 Simultaneous Saccharification and Fermentation (SSF)

**FIGURE 7:**
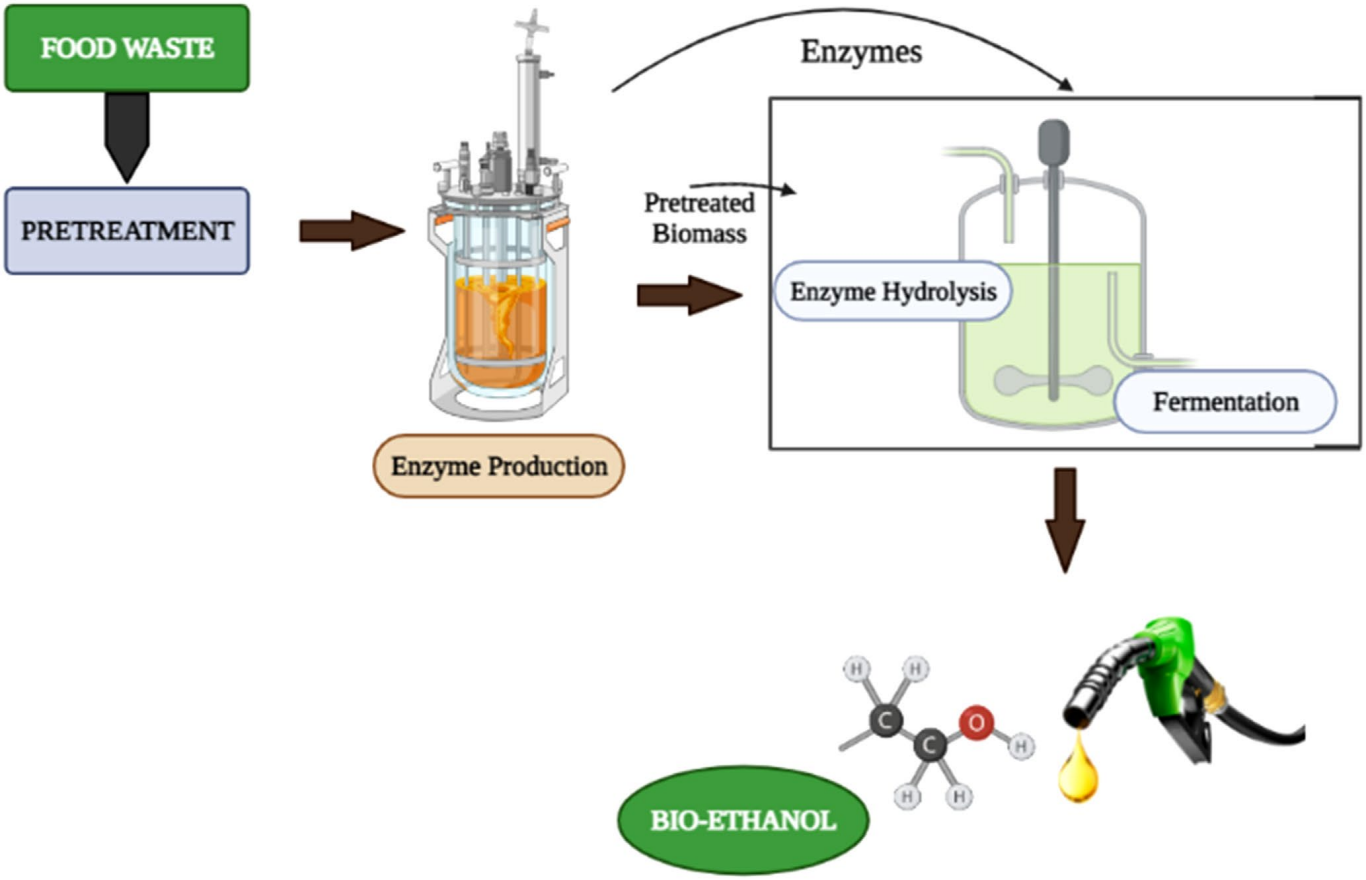
Simultaneous saccharification/hydrolysis and fermentation for bioethanol production.

A saccharifying enzyme and a yeast‐based fermenting microbe are both introduced into a reactor at the same time in the SSF process, as shown in Figure 7. Since hydrolysis and fermentation happen concurrently in the same reactor, the procedure strives to be economical, but it is challenging to create the ideal conditions for both processes at once (Kucharska et al. 2018; Olivieri et al. 2021). For instance, yeast grows best at 30°C, but enzyme activity begins at 50°C. As a result, the need for synergy turns into a challenge that can be partially overcome by employing thermostable yeast (Schmerling et al. 2022; Abushahab et al. 2024). The normal inhibition of cellulase activity can be avoided since the hydrolysate is used concurrently for fermentation, and the microbe uses the sugars generated during the process for both growth and bioethanol production, enhancing bioethanol production.

#### 6.1.3 Simultaneous Saccharification and Co‐Fermentation (SSCF)

Considering that *Saccharomyces cerevisiae* typically cannot ferment pentose sugar, simultaneous saccharification and co‐fermentation is a practical method for producing bioethanol. Enzymatic hydrolysis of pretreated biomass occurs concurrently with the co‐fermentation of hexose and pentose (primarily glucose and xylose) by genetically modified yeast. The benefits of SSCF include reduced cost, increased bioethanol yield, and speedier processing. Additionally, SSCF increases the ratio of xylose to glucose since most bacteria can ingest xylose and reduces the inhibitory effects of high sugar concentrations on enzymatic hydrolysis (Li, Chen, et al. 2019; Sun et al. 2021). Controlling temperature presents a considerable challenge in SSCF, though. While yeast or bacteria normally function at temperatures between 30°C and 37°C, the ideal temperature for enzymatic activity during the saccharification process is around 50°C (Zhu et al. 2020). Consequently, it is crucial to take into account temperature regulation in this procedure.

#### 6.1.4 Consolidated Bioprocessing

**FIGURE 8:**
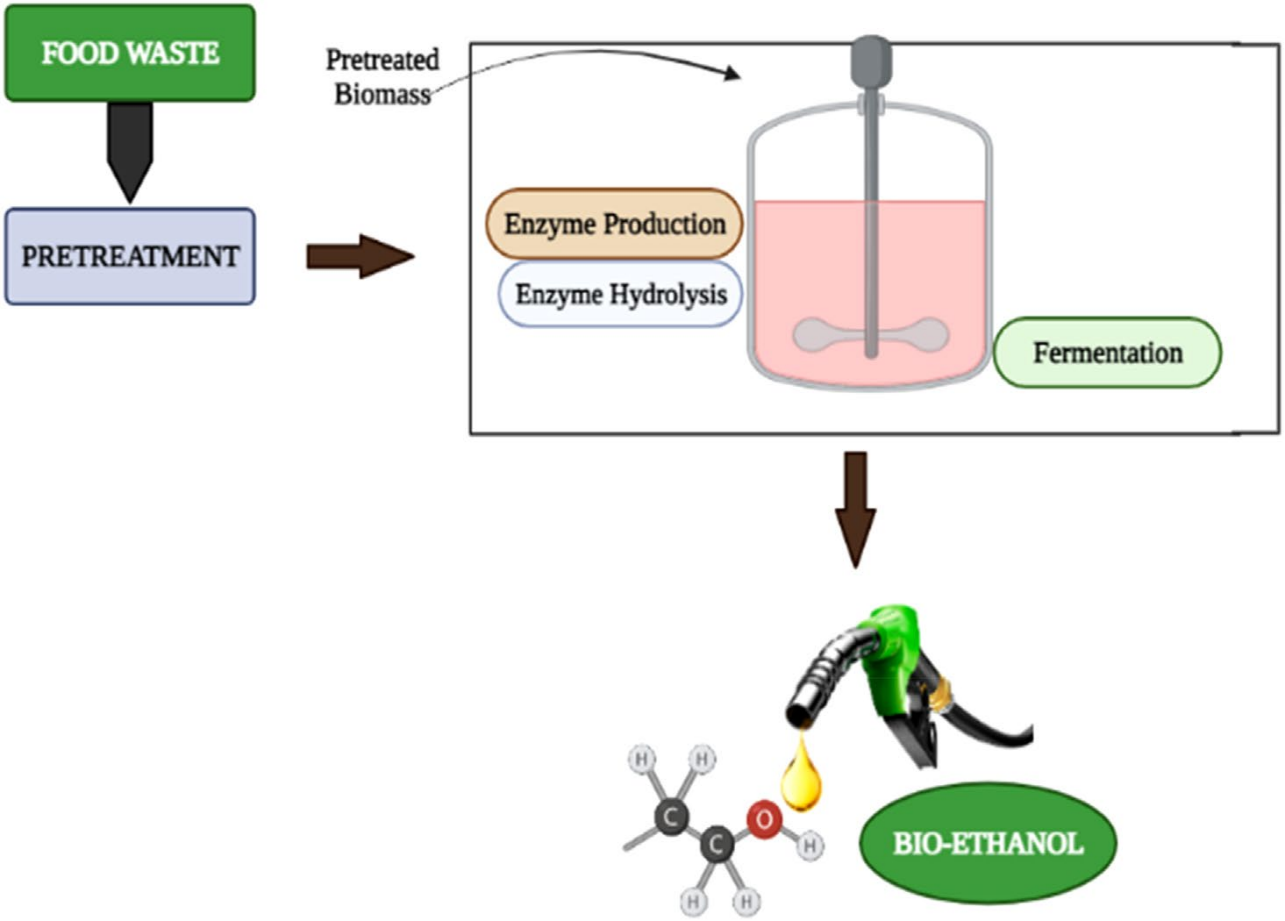
Consolidated bioprocessing for bioethanol production.

Consolidated Bioprocessing (CBP), also known as Single‐Step Bioprocessing or Single‐Pot Bioprocessing, is another bioprocessing approach in addition to SHF and SSF. It offers a more effective alternative to previous bioprocessing techniques. This method uses a single microorganism or a group of microorganisms to carry out the three crucial steps of enzyme production, saccharification (the conversion of complex carbohydrates into simple sugars), and bioethanol production in a single bioprocessing reactor from biomass waste, food waste, or lignocellulosic biomass (Mazzoli 2021; Singhania et al. 2022). Figure 8 shows the consolidated bioprocessing for bioethanol production.

A viable option for waste management and the production of sustainable energy is the use of food waste for bioethanol. Starch, protein, and fat‐rich food waste make an excellent carbon source for fermentative biofuel production. Enzymatic hydrolysis, fermentation, distillation, and pretreatment are all steps in the synthesis of bioethanol. Different bioprocessing techniques, including SHF, SSF, SSCF, and CBP, are crucial for increasing productivity and sustainability. These methods have various benefits, such as better bioethanol yields, lower costs, and quicker processing times, which help to produce bioethanol from food waste in a greener and more effective manner.

Enzymatic hydrolysis coupled with optimized fermentation typically achieves 60%–90% of the theoretical ethanol yield, with starchy and sugary food wastes often performing at the higher end of this range (Dharma Patria et al. 2022; Chen et al. 2022; Samantaray et al. 2024). The purity of the produced bioethanol is primarily determined during the distillation and dehydration stages. Most studies indicate that food‐waste‐derived ethanol can be upgraded to fuel‐grade purity using conventional distillation followed by molecular sieves or pervaporation membranes (Peng et al. 2021; Kujawska et al. 2024; Jain and Kumar 2024; Sharma et al. 2026). These findings demonstrate that bioethanol derived from food waste is comparable in quality to ethanol produced from other biomass feedstocks and can meet international standards for biofuel applications.

## 7 Conclusion

Growing volumes of food waste present both an environmental burden and an untapped opportunity for renewable‐fuel production. Evaluating its physicochemical properties shows that food waste contains substantial amounts of readily degradable carbohydrates and high moisture levels, making it a suitable second‐generation feedstock for sustainable bioethanol production. Effective pretreatment is essential for improving substrate accessibility, and while physical and chemical methods enhance sugar release, enzymatic and biological pretreatments offer greater selectivity and reduced environmental impact. The efficiency of downstream hydrolysis and fermentation is strongly influenced by these pretreatment choices, with fermentation serving as the key yield‐determining stage. Among the various bioprocessing configurations, SSF and SSCF demonstrate superior conversion efficiencies and shorter overall processing times compared with SHF, while consolidated bioprocessing offers long‐term promise for simplifying operations and reducing costs. Collectively, the findings indicate that integrating suitable pretreatment strategies with optimized fermentation can significantly enhance bioethanol yields while supporting more sustainable and circular approaches to food‐waste management.

## Author Contributions

**S.S.**, **S.S.**, **G.S.**, **S.K.A.**, **R.R.M.** and **B.R**.: conceptualization, methodology, supervision, investigation, data curation, writing – original draft, writing – review and editing. **A.P.**, **R.S.**, **K.R.**, **S.C.** and **S.W.C**.: writing – review and editing. **S.K.A.**, **R.R.M.** and **B.R**.: project administration and funding.

## Funding

We are thankful for the support provided by UCSI University for the support provided by the Center of Excellence for Research, Value Innovation and Entrepreneurship (CERVIE) and Research Excellence and Innovation Grant (REIG) with code REIG‐FPS‐2025/038. This work was supported by the Korea Institute of Energy Technology Evaluation and Planning (KETEP) and the Ministry of Trade, Industry & Energy (MOTIE) of the Republic of Korea (No. RS‐2023‐00255939).

## Ethics Statement

The authors have nothing to report.

## Consent

The authors have nothing to report.

## Conflicts of Interest

The authors declare no conflicts of interest.

## Data Availability

The data that support the findings of this study are available from the corresponding author upon reasonable request.
